# Coronary artery bypass grafting in patients with hematological neoplasms

**DOI:** 10.3389/fcvm.2025.1697389

**Published:** 2025-12-17

**Authors:** Lixue Zhang, Simeng Zhang, Hao Jiang, Jian Liu, Qing Gao, Bo Lian, Zeye Liu, Yi Shi

**Affiliations:** 1 Department of Cardiac Surgery, Peking University People’s Hospital, Beijing, China; 2 Department of Hematology, Peking University People’s Hospital, Beijing, China; 3 Department of Cardiology, Peking University People’s Hospital, Beijing, China

**Keywords:** CABG, hematological neoplasms, multidisciplinary management, coronary artery disease, preoperative blood cell level

## Abstract

**Background:**

Coronary artery bypass grafting (CABG) in patients with concurrent coronary artery disease (CAD) and hematological neoplasms presents unique challenges due to immunosuppression, hematologic dysfunction, and coagulopathy. This study aimed to assess the safety and feasibility of CABG in this population and to evaluate factors influencing prognosis.

**Methods:**

This retrospective study included 41 patients with CAD and hematological neoplasms who underwent CABG between 2018 and 2023. Hematological neoplasms were classified into seven categories, and patients were stratified by hematological disease status: stable, disease-free, or progressive. Key perioperative outcomes, graft patency, and survival data were analyzed. Cox regression models identified independent predictors of prognosis.

**Results:**

Of the 41 patients, 28 (68.3%) were stable, 10 (24.4%) were disease-free, and 3 (7.3%) were progressive. The median preoperative platelet count was 143 × 10^9^/L, with 8 patients presenting counts <50 × 10^9^/L requiring preoperative platelet transfusions. Postoperative transfusion rates for packed red blood cells (PRBCs), fresh frozen plasma (FFP) and platelets were 51.2%, 39.0%, and 12.2%, respectively. The median operation time was 210 min, with 87.8% undergoing off-pump CABG. Graft patency at discharge was 92.3%. Major infections occurred in 4.9% of patients, and 9 (22%) deaths were recorded during follow-up, 8 due to hematological progression and 1 due to myocardial infarction. Cox regression identified preoperative blood cell levels as independent predictors of survival (*p* < 0.05), while CABG-related factors showed no significant association (*p* > 0.05).

**Conclusions:**

CABG can be performed safely in most patients with stable hematological neoplasms, with no perioperative mortality, providing an opportunity for further hematological treatments. Preoperative blood cell levels significantly influence prognosis, underscoring the importance of multidisciplinary management. Larger studies are needed to validate these findings and refine treatment strategies.

## Introduction

Coronary artery disease (CAD) is a major cardiovascular condition affecting populations worldwide and recognized as the primary cause of mortality (1). The contemporary management of CAD encompasses primary and secondary prevention through medical therapy and revascularization when deemed appropriate based on current evidence-based guidelines (2). Coronary artery bypass grafting (CABG) is a principal revascularization approach, particularly favored for individuals with complex multiple vessel disease, diabetes, reduced left ventricular function, and contraindications to dual anti-platelet therapy (DAPT) (2). It is crucial to comprehensively evaluate candidates for CABG to determine surgical risk (3, 4).

Hematological neoplasms constitute a heterogeneous group of disorders originating from bone marrow and the lymphatic system. These disorders are broadly categorized into leukemia, lymphoma, plasma cell neoplasms, myelodysplastic syndromes (MDS), and myeloproliferative neoplasms (MPN). The classification is intricate, influenced by distinct clinicopathological features, prognosis, and therapeutic protocols (5). Patients with hematological neoplasms often exhibit immune dysregulation, hematopoietic dysfunction, and coagulopathy.

In recent years, an increasing number of elderly patients at our center has been diagnosed with both CAD and hematological neoplasms concurrently. Managing CAD is particularly challenging because of coexisting immunologic and hematologic disorders. Similarly, treating hematological diseases is fraught with significant risk in the setting of myocardial ischemia. This raises uncertainty about the optimal therapeutic strategy for individuals necessitating revascularization for CAD with concurrent hematological neoplasms. This study aimed to assess the safety and feasibility of CABG in this population and to evaluate factors influencing prognosis.

## Method

We performed a retrospective review of patients undergoing CABG at our center between January 2018 and December 2023 who had concomitant CAD and hematological neoplasms. The inclusion criteria for this study were: (1) patients who underwent isolated coronary artery bypass grafting (CABG) at our center between January 2018 and December 2023; (2) patients who also had a concurrent hematological neoplasm. The exclusion criterion were: (1) patients with missing perioperative or follow-up data; (2) patients underwent concomitant cardiac procedures other than CABG; (3) emergent or salvage surgery. The hematological neoplasms were classified into seven categories: acute myeloid leukemia (AML), acute lymphocytic leukemia (ALL), myelodysplastic syndromes (MDS), lymphoma, chronic myeloid leukemia (CML), plasma cell neoplasms, and myeloproliferative neoplasms (MPN). In total, 41 eligible patients were enrolled in this retrospective study.

To enhance treatment outcomes, a multidisciplinary team consisting of cardiac surgeons, cardiologists, and hematologists was convened. CABG recommendations were based on patients' coronary angiography, symptoms of myocardial ischemia, and comprehensive health assessment. Senior surgeons devised personalized surgical plans for revascularization, including decisions regarding the type of incision, use of cardiopulmonary bypass (CPB), choice of conduit, target vessel, and grafting strategy. Hematological neoplasms were diagnosed according to the 2016 WHO classification. Each hematological neoplasm was diagnosed based on morphological and pathological criteria. Based on their hematological status, patients were stratified into three categories: (1) disease-free status: patients who have undergone systemic treatment and achieved complete remission; (2) progressive status: patients who require urgent systemic treatment due to poor prognosis; and (3) stable status: patients in a controlled state but do not yet require tumor-specific treatment. The multidisciplinary team formulated an integrated treatment protocol, including cardiac and hematological interventions, for each patient, considering disease status, treatment risks, and patient preferences.

Transit time flowmetry (TTFM) was employed for intraoperative graft evaluation, and re-anastomosis was performed if TTFM results were unsatisfactory. Unless contraindicated by renal dysfunction, contrast agent allergies, or patient preference, postoperative computed tomography (CT) angiography was performed to assess graft patency prior to discharge. Antithrombotic treatment, including antiplatelet and anticoagulation therapy, was initiated for eligible patients. The treatment strategy was tailored to each patient's needs, considering their clinical status, platelet counts, and coagulation profile.

The grafting strategy was documented and encompassed the surgical approach, use of CPB, types and numbers of target vessels, type of conduit, and the utilization of sequential grafting and composite conduits. Additional perioperative data encompassed operation time, pre- and postoperative transfusion of packed red blood cells (PRBCs), fresh frozen plasma (FFP), and platelets, total drainage, ventilation duration, ICU stay duration, and postoperative hospital stay length.

Data on hematological conditions were retrospectively reviewed, including disease status and stage, hematological therapy administered before and after surgery, perioperative complete blood cell counts (encompassing leukocyte, hemoglobin, and platelet counts), and perioperative coagulation function.

The primary endpoint was postoperative adverse events comprising in-hospital mortality, postoperative myocardial infarction (MI), stroke, and repeat revascularization. This study additionally examined bleeding and thrombotic complications, infectious events, and follow-up outcomes. Major infection was defined as deep incisional surgical site infection, mediastinitis, infectious myocarditis or pericarditis, endocarditis, pneumonia, empyema, or bloodstream infection. During the follow-up, we regularly contacted patients or their families by phone calls to obtain updates on their disease status. The last follow-up date was December 31, 2023. All-cause mortality was used in this study.

All the data were anonymized during the process in this retrospective study, in accordance with the regulations of ethics committee of Peking University People's Hospital. All procedures complied with the Declaration of Helsinki. We use a retrospective design due to the relatively small number of patients with both coronary artery disease and hematological neoplasms, as well as the diversity of disease types, which makes random allocation difficult. For missing data, we used multiple imputation to handle it, reducing the risk of sample size reduction and bias. To control for potential confounding factors, we included age, gender, and underlying diseases in the Cox regression model to adjust for their effects on survival. Given the nature of retrospective studies, various biases are inevitable; to reduce selection bias, we strictly adhered to inclusion and exclusion criteria, ensuring the representativeness of the sample. To minimize information bias, we used electronic medical record data and performed rigorous data verification.

Continuous variables are reported as mean ± standard deviation (SD) or median with interquartile range (IQR). Comparisons were conducted using Student's *t*-test or Mann–Whitney *U*-test, as appropriate. Categorical variables were expressed as percentages. An overall survival analysis was performed, and a Cox proportional hazards regression model was utilized to evaluate the influence of potential risk factors on survival. The Schoenfeld residuals test was used to verify the proportional hazards assumption. A *p*-value of <0.05 was considered statistically significant. Statistical analyses were conducted with SPSS Statistics 25 (IBM Corp., Armonk, NY, USA).

## Results

**Figure 1:**
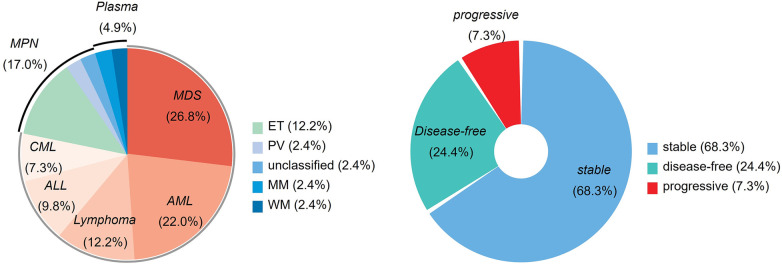
The proportion of hematological neoplasms and hematological status. AML, acute myeloid leukemia; ALL, acute lymphocytic leukemia; CML, chronic myeloid leukemia; MDS, myelodysplastic syndromes; MPN, myeloproliferative neoplasms; Plasma, plasma cell neoplasms; ET, essential thrombocythemia; PV, polycythemia vera; MM, multiple myeloma; WM, Waldenström's Macroglobulinemia.

This study enrolled 41 eligible patients consecutively. The baseline characteristics are summarized in Table 1. The hematological neoplasms were distributed as follows: MDS (11), AML (9), MPN (7), lymphoma (5), ALL (4), CML (3), and plasma cell neoplasms (2). Among them, 28 patients (68.3%) were classified as stable, 10 patients (24.4%) were in remission, and 3 patients (7.3%) had disease progression (Figure 1, Supplementary Table 1).

**Table 1:** Baseline characteristics of the patients in this study.

Variable	Value (*N* = 41)
Age (years)	57.7 ± 10.6
Male, *n* (%)	33 (80.5)
Body mass index (kg/m^2^)	24.5 (22.4–26.2)
Smoke, *n* (%)	25 (61.0)
Hypertension, *n* (%)	20 (48.8)
Diabetes mellitus, *n* (%)	19 (46.3)
Dyslipidemia, *n* (%)	10 (24.4)
Peripheral artery disease, *n* (%)	3 (7.3)
Cerebrovascular disease, *n* (%)	4 (9.8)
Renal dysfunction, *n* (%)	1 (2.4)
Stable CAD, *n* (%)	10 (24.4)
Non-ST elevation ACS, *n* (%)	28 (68.3)
STEMI, *n* (%)	3 (7.3)
Previous MI, *n* (%)	6 (14.6)
Previous PCI, *n* (%)	6 (14.6)
NYHA class III/IV, *n* (%)	5 (12.2)
LVEF (%)	62.9 ± 9.6
LVEDD (mm)	50.7 ± 5.4
Single-vessel disease, *n* (%)	20 (48.8)
Three-vessel disease, *n* (%)	13 (31.7)
Left main disease, *n* (%)	7 (17.1)

Data are *n* (%), mean ± SD or median (IQR).

CAD, coronary artery disease; ACS, acute coronary syndrome; STEMI, ST elevation myocardial infarction; MI, myocardial infarction; PCI, percutaneous coronary intervention; LVEF, left ventricular ejection fractions; LVEDD, left ventricular end diastolic diameter.

**Figure 2:**
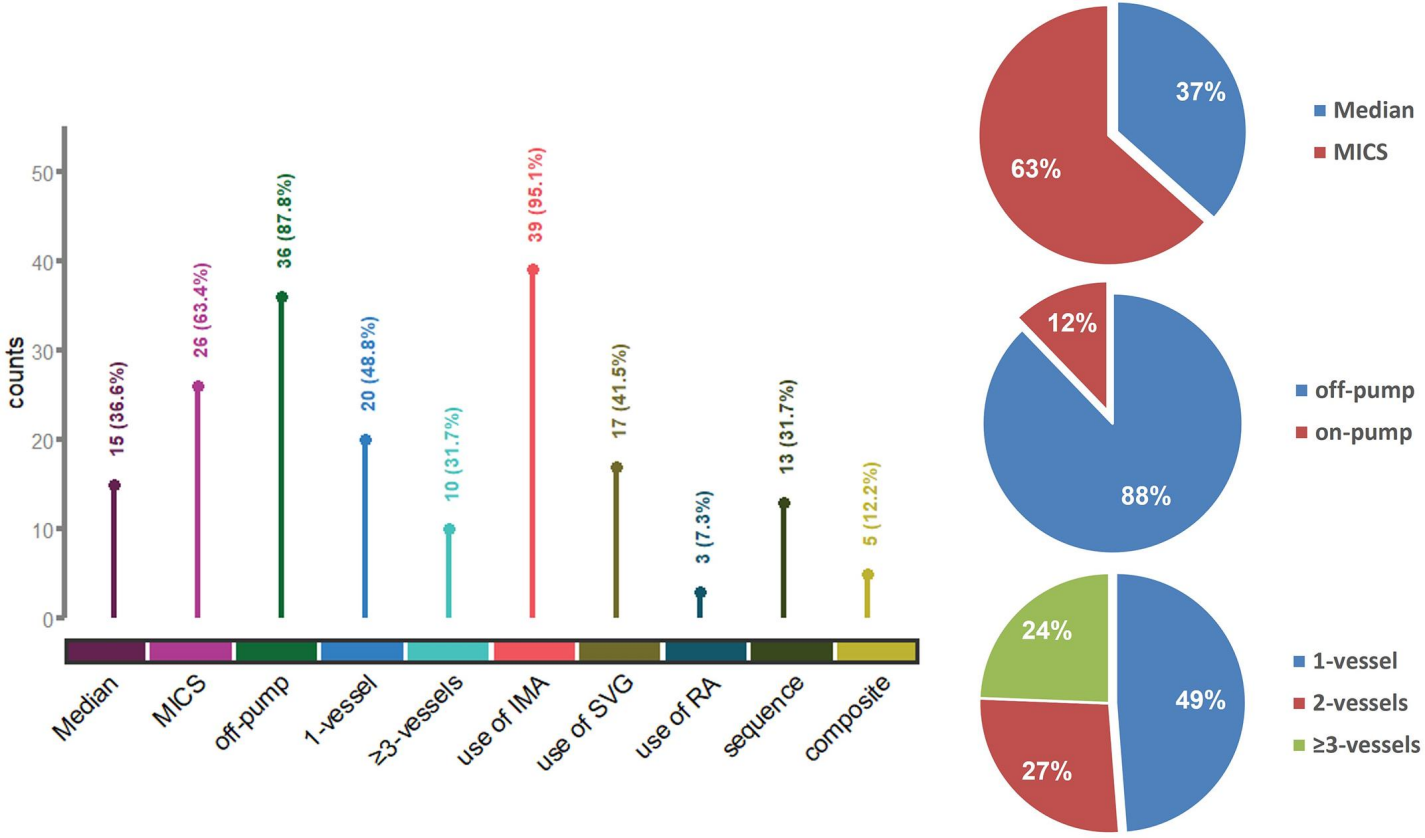
Surgical information and characteristics of the bypass procedure. Median, median sternotomy; MICS, minimally invasive coronary surgery; IMA, internal mammary artery; SVG, saphenous vein graft; RA, radial artery; sequence, use of sequential grafting; composite, use of composite conduits.

Surgical information is detailed in Supplementary Table 2. The majority of patients (87.8%) underwent off-pump CABG, avoiding extracorporeal circulation. Minimally invasive coronary surgery (MICS) via a left anterior thoracotomy was conducted in 26 patients (63.4%). Nearly half the patients (48.8%) underwent single-vessel bypass (Figure 2). The median operation time from incision to closure was 210 min.

**Figure 3:**
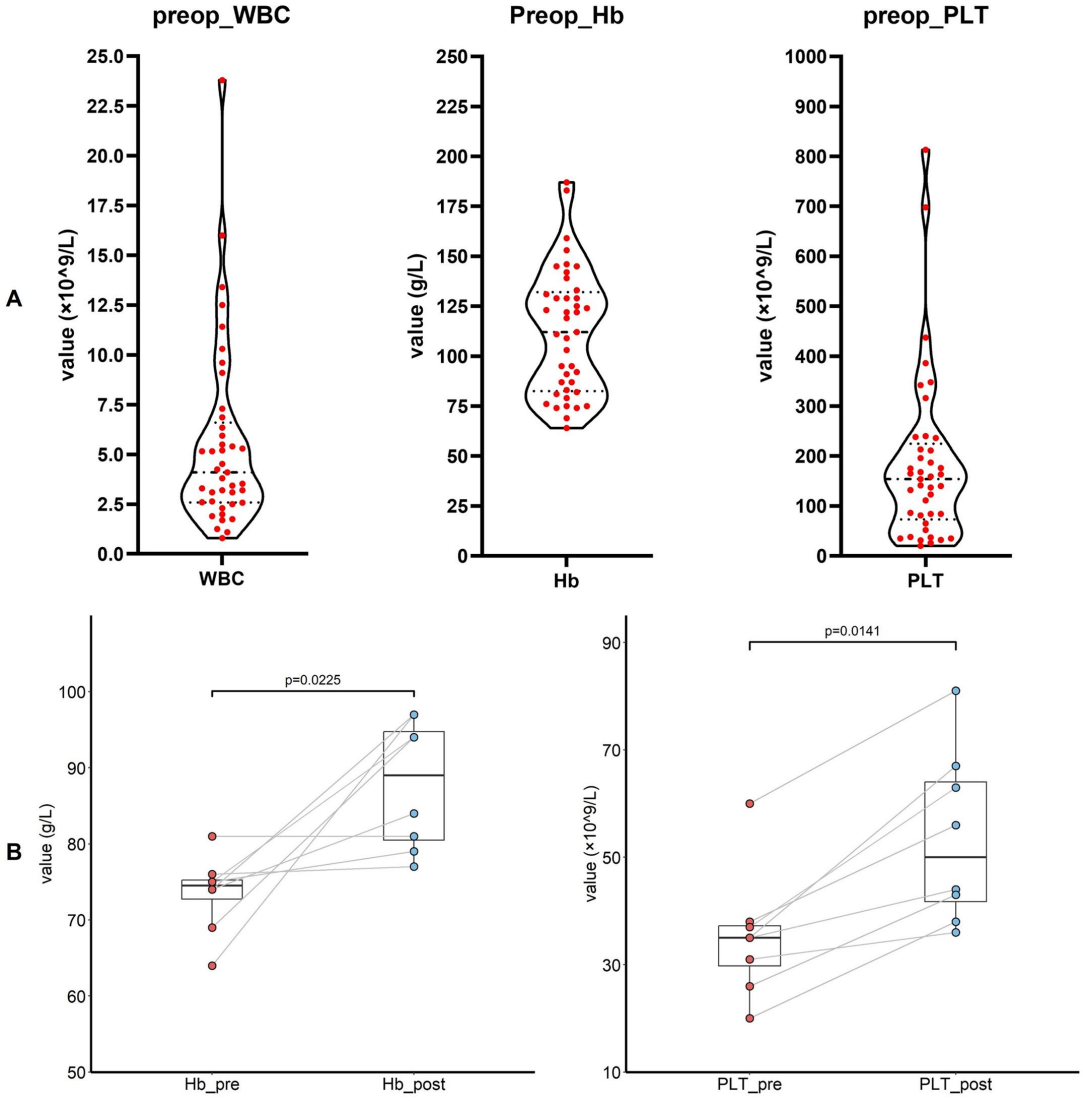
**(A)** The preoperative level of white blood cell, hemoglobin and platelet (*n* = 41 in each panel). **(B)** The preoperative level of hemoglobin and platelet before and after the transfusion. WBC, white blood count; Hb, hemoglobin; PLT, platelet.

Figure 3A illustrates the distribution of preoperative white blood cell counts, platelet levels, and hemoglobin concentrations. Preoperative transfusions of PRBCs and platelets were administered in eight cases each to elevate these parameters (Figure 3B). Postoperatively, more than half of the patients (51.2%) received PRBC transfusions; 39% and 12.2% of patients received FFP and platelet transfusions, respectively. Twenty-nine patients (70.7%) received antiplatelet therapy, with 16 patients on single-agent therapy and 13 patients on dual-antiplatelet therapy (Supplementary Table 3).

**Figure 4:**
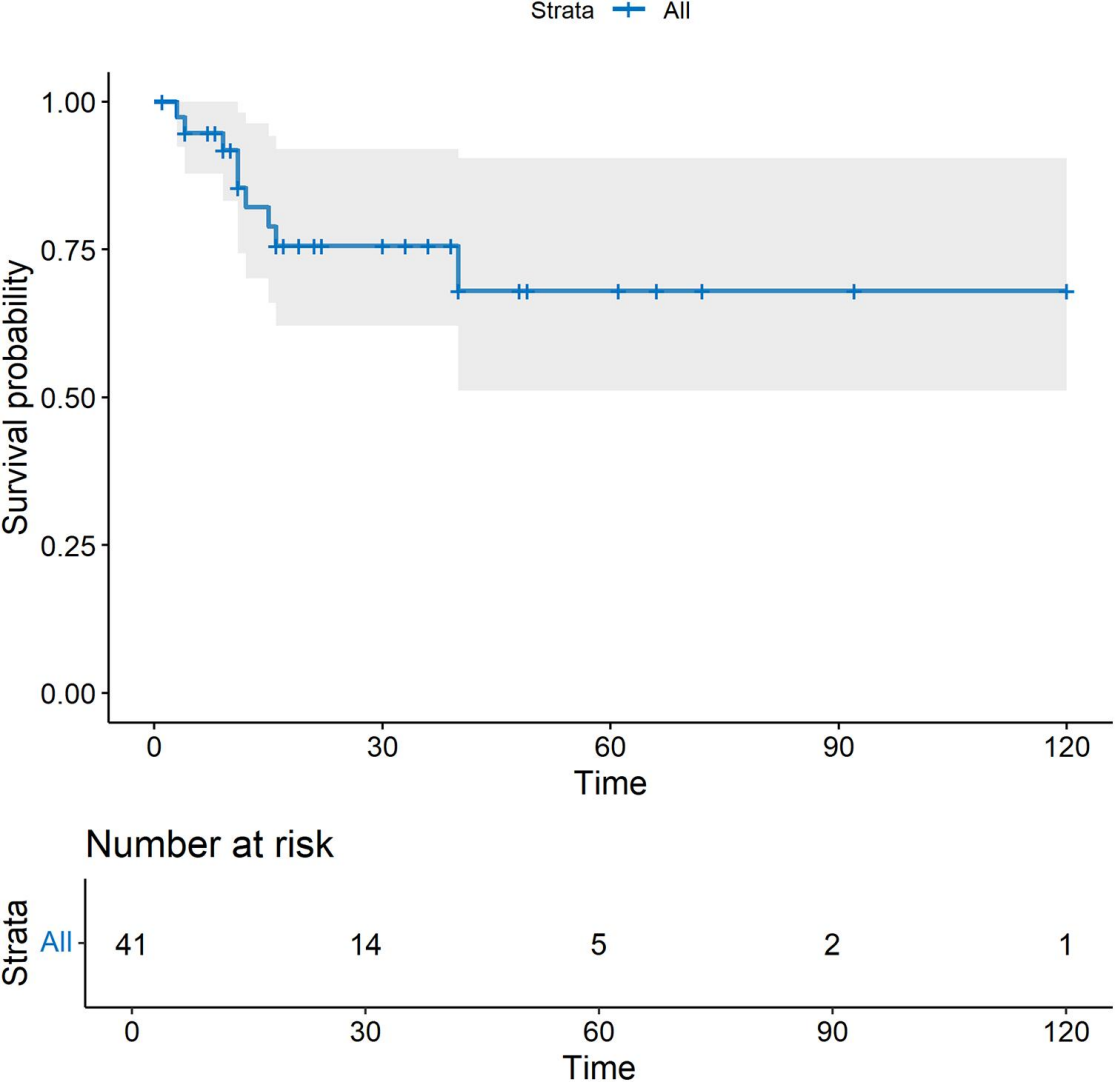
The survival curve after CABG. All-cause mortality was used.

**Figure 5:**
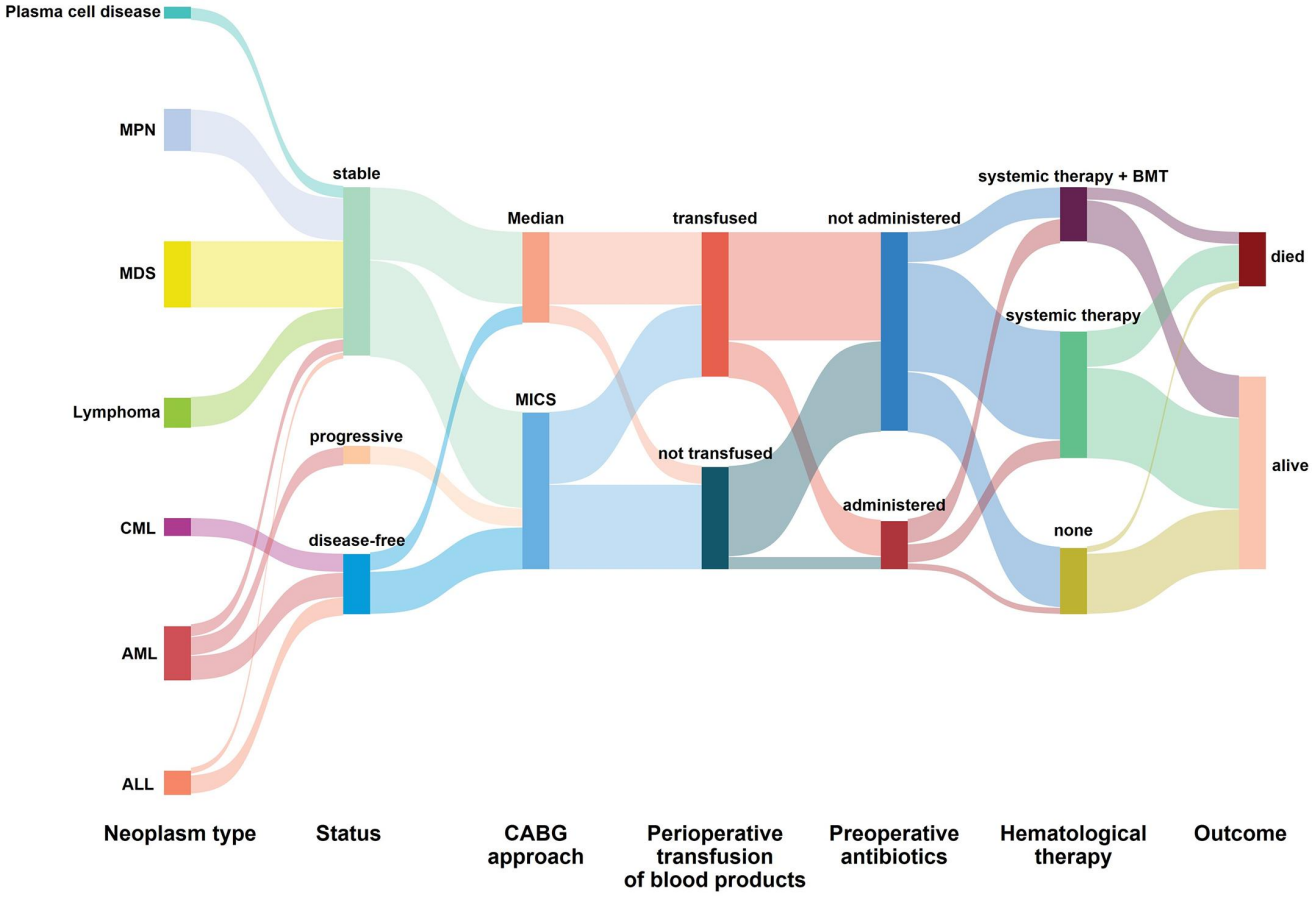
The information regarding neoplasms, therapeutic strategies, and outcomes. AML, acute myeloid leukemia; ALL, acute lymphocytic leukemia; CML, chronic myeloid leukemia; MDS, myelodysplastic syndromes; MPN, myeloproliferative neoplasms; MICS, minimally invasive coronary surgery; Median, median sternotomy; BMT, bone marrow transplantation.

**Figure 6:**
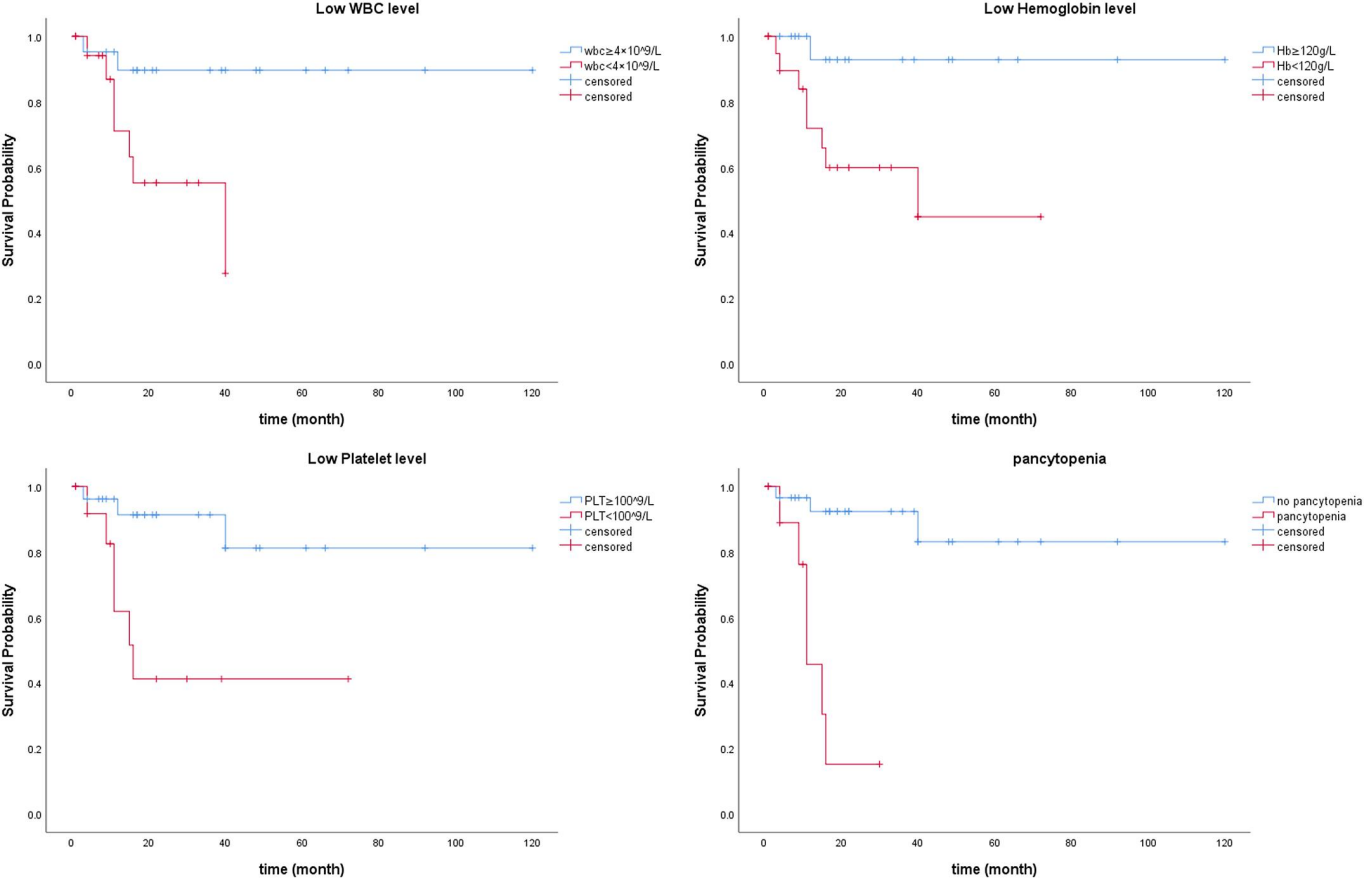
Kaplan–Meier curves illustrating the impact of relevant variables on prognosis.

No major adverse events occurred during the perioperative period (Table 2). In this study, one patient had a mild contrast agent allergy preoperatively, and another refused to complete the scan, so these two patients did not undergo coronary assessment before discharge. The short-term graft patency rate, based on CT angiographic assessment, was 92.3%. All patients with graft occlusion detected were asymptomatic and did not undergo further intervention. All the 41 patients completed the follow-up. The duration spanned 1–120 months and had a median duration of 17 months (Figure 4). Landmark survival estimates were calculated. The estimated 1-year survival was 82.2% (95% CI: 75.5%–88.9%), and the 40-month survival was 68.0% (95% CI: 58.1%–77.9%). Nearly three-quarters of the patients received systemic hematological therapies after CABG, and one-fourth underwent bone marrow transplantation (Table 2). Nine patients died, eight had progression or relapse of their hematological neoplasm before death, with 3 dying from multiple organ failure, 3 from severe infections after chemotherapy, 1 from severe infection following bone marrow transplantation failure, and 1 from a cerebral hemorrhage; the remaining patient, in stable hematological condition, died from cardiogenic shock following acute myocardial infarction. The survival times ranged from 3 to 40 months post-discharge. Details of these nine patients are provided in Supplementary Table 4. The information regarding neoplasms, therapeutic strategies, and outcomes is presented in Figure 5. In the univariate Cox regression analysis, sex, preoperative blood cell levels, postoperative transfusion of PRBCs, postoperative antiplatelet therapy, and AML showed significant associations with survival outcomes (*p* < 0.05) (Table 3). Kaplan–Meier curves illustrated the impact of relevant variables on prognosis (Figure 6).

**Table 2:** The outcomes and follow-up information.

Variable	Value (*N* = 41)
Drainage (mL)	903.7 ± 495.3
Ventilation time (h)	6.0 (4.0–13.5)
ICU length of stay (h)	27.0 (22.0–65.0)
Postoperative length of stay (days)	8.0 (7.0–10.0)
In-hospital death	0
Stroke	0
Postoperative MI	0
Repeated revascularization	0
Bleeding and clotting events	0
Major infection, *n* (%)	2 (4.9)
Patent graft at discharge, (*N* = 39) (%)	36 (92.3)
Postoperative systemic therapy, *n* (%)	30 (73.2)
Postoperative BMT, *n* (%)	9 (22.0)

Data are *n* (%), mean ± SD or median (IQR).

MI, myocardial infarction; BMT, bone marrow transplantation.

**Table 3:** Univariate Cox regression analysis of survival.

Characteristics	HR	95% CI	*p* Value
Age	1.00	0.94–1.06	0.996
Body mass index	0.86	0.67–1.10	0.236
male	0.22	0.06–0.85	0.027
Smoke	1.11	0.27–4.47	0.887
Hypertension	1.25	0.34–4.65	0.741
Diabetes mellitus	1.27	0.34–4.74	0.725
Non-ST elevation ACS	1.95	0.49–7.85	0.345
NYHA class III/IV	1.82	0.38–8.79	0.458
LVEF	0.962	0.90–1.03	0.243
Preop-WBC	0.68	0.46–0.99	0.042
Preop-Hb	0.97	0.95–1.00	0.043
Preop-PLT	0.99	0.98–1.00	0.019
Postop-PRBCs	9.40	1.17–75.38	0.035
Postop-FFP	1.30	0.35–4.86	0.694
Postop-Platelet	3.28	0.66–16.29	0.147
MICS	2.11	0.44–10.16	0.352
On-pump CABG	1.15	0.14–9.21	0.897
Grafts <3	1.73	0.36–8.34	0.496
Sequential grafting	1.40	0.37–5.24	0.619
Composite conduits	3.14	0.78–12.58	0.107
Length of stay (day)	0.70	0.46–1.06	0.089
Preop-APT	2.08	0.51–8.48	0.307
Preop-LMWH	7.76	0.97–62.26	0.054
Postop-APT	0.18	0.05–0.70	0.013
Neoplasms			
AML	3.80	1.01–14.26	0.048
ALL	1.52	0.19–12.40	0.695
MDS	2.15	0.53–8.80	0.286
Lymphoma	0.79	0.10–6.35	0.826
Status			
Stable	0.52	0.14–1.96	0.337
Disease-free	1.20	0.25–5.78	0.824
Progressive	2.50	0.52–12.10	0.254
Systemic therapy	2.56	0.32–20.47	0.376
Bone marrow transplantation	1.41	0.28–7.03	0.673
Patent grafts	0.71	0.09–5.69	0.747

ACS, acute coronary syndrome; LVEF, left ventricular ejection fractions; WBC, white blood cell; Hb, hemoglobin; PLT, platelet; PRBCs, packed red blood cells; FFP, fresh frozen plasma; MICS, minimally invasive coronary surgery; CABG, coronary artery bypass grafting; APT, anti-platelet therapy; LMWH, low-molecular-weight heparin; AML, acute myeloid leukemia; ALL, acute lymphocytic leukemia; MDS, myelodysplastic syndromes; grafts <3, single- or double- vessel bypassed.

## Discussion

In recent years, an increasing number of patients with CAD and hematological neoplasms undergoing CABG have been observed at our center. These patients typically presented with preexisting blood diseases while presenting with myocardial infarction at our center. Only a minority of patients were concurrently diagnosed with both CAD and hematological neoplasms. The mechanisms underlying the association between CAD and hematological neoplasms are not yet fully elucidated.

Clonal hematopoiesis (CH), characterized by the presence of somatic mutations in hematopoietic stem cells that result in mutant leukocytes, is recognized as a critical risk factor for hematological neoplasms (6, 7). Many of the mutations commonly observed in CH are implicated in the development of AML (8), MDS (9–11), myeloproliferative neoplasms (12), and certain lymphomas (13, 14), which confer a 10-fold elevated relative risk for these neoplasms over time (15, 16). Recent evidence indicates that CH is associated with accelerated atherosclerosis and a heightened risk of atherosclerotic cardiovascular events in humans (17). This suggests that CH promotes both hematological neoplasms and the progression of cardiovascular disease. Data on CH mutations, however, were unavailable for the majority of patients in this cohort, making the mechanistic speculation hypothesis-generating. Further research is needed to elucidate the association between CAD and blood diseases. Additionally, aging, inflammation, and physiological and biochemical mechanisms may contribute to the risk of developing both conditions.

Traditionally, patients with hematological neoplasms were deemed unsuitable candidates for CABG due to poor prognosis, high risks of bleeding and infection, and the complexity of multi-organ involvement. However, with an increasing number of patients in this category presenting to our center, the feasibility of CABG has been increasingly investigated in recent years. The indications for surgery in CAD patients include persistent symptoms refractory to medical therapy and the potential for improved prognosis (18). For patients with blood diseases, CABG also aims to address contraindications for various hematological treatments, including chemotherapy, targeted therapy, immunotherapy, and bone marrow transplantation. In this study, 87.8% of the CABG procedures were off-pump, a relatively high proportion, reflecting both the surgical preference at our center and concerns regarding the potential impact of cardiopulmonary bypass on the hematologic system. It is also worth noting that the choice of surgical approach, either minimally invasive or conventional median sternotomy, was determined based on the surgeon's comprehensive assessment of the patient's individual conditions, cardiac function, anatomical factors, and the number of grafts required.

Most patients in this study were classified as stable or disease-free, maintaining hematological and clinical stability without pharmacological intervention, particularly in cases of myeloproliferative neoplasms and myelodysplastic syndromes. Stability was achieved through systemic therapies in patients with lymphoma, multiple myeloma, and select acute leukemia cases. CABG was performed to address cardiac contraindications and reduce therapeutic risks, with progressive disease requiring effective hematological management prior to surgery (19). For three patients with advanced-stage AML, CABG was performed using minimally invasive techniques due to severe coronary stenosis and ischemia, despite hematological treatment contraindications. No perioperative adverse events occurred, and all three received subsequent systemic therapies, with one undergoing bone marrow transplantation. Two patients died from disease recurrence 16 and 40 months post-surgery.

Among the 41 patients included in this study, 9 deaths were recorded during the follow-up period. These patients included 4 with AML, 1 with ALL, 3 with MDS, and 1 with lymphoma, all characterized by aggressive malignant behavior and poor prognostic outcomes. Eight of the deceased patients underwent systemic therapy for hematological neoplasms, and 2 received bone marrow transplantation. Among the 9 deaths, 8 experienced hematological disease progression, while 1 was due to myocardial infarction. Analysis of surgical data revealed that 7 of these patients underwent minimally invasive CABG, with 4 receiving single-vessel bypass grafting and only 2 undergoing three or more grafts. We believe that compared to cardiovascular diseases, the malignant progression of hematological neoplasms may have a greater impact on prognosis. However, the role of cardiovascular status in exacerbating the outcome should not be overlooked, which further emphasizes the importance of postoperative cardiovascular management in this patient population.

Cox regression analyses identified preoperative blood cell levels, rather than coronary artery disease severity or CABG-related variables, as independent predictors of prognosis. The absence of a significant association between the type or status of hematological neoplasms and prognosis in Cox regression analysis may reflect the limited sample size. Nonetheless, these findings suggest that hematological neoplasms may have a more substantial impact on prognosis compared to coronary artery disease in this patient population. Further investigations with larger cohorts are warranted to validate these observations and refine prognostic assessment in this unique population.

The primary concerns included bleeding and clotting disorders. Eight patients with platelet counts <50 × 10^9^/L received preoperative transfusions, with a median platelet count of 143 × 10^9^/L at surgery. Postoperative transfusion rates for PRBCs, FFP, and platelets were 51.2%, 39.0%, and 12.2%, respectively—higher than typical. Despite this, bleeding and clotting events were rare. Perioperative antiplatelet therapy was individualized based on ischemic risk and platelet counts (20, 21). DAPT was used for acute coronary syndrome or multivessel disease if platelet counts exceeded 80 × 10^9^/L. A single antiplatelet agent was prescribed for counts of 50–80 × 10^9^/L, while antiplatelet therapy was withheld for counts <50 × 10^9^/L due to bleeding risk.

Seven patients with agranulocytosis received prophylactic antibiotics preoperatively. Postoperatively, all patients received antibiotics, primarily second-generation cephalosporins or culture-guided regimens. Major infections occurred in 2 patients (4.9%), consistent with reported rates (22). Immunosuppression in hematological neoplasms necessitates proactive and prolonged antibiotic use, along with isolation measures and strict sterilization protocols.

### Limitation

This study has certain limitations. Firstly, the inherent biases of single-center retrospective studies are difficult to fully eliminate. Future prospective studies with more rigorous designs can further validate these findings. Secondly, the small sample size, reflecting the rarity of patients with both coronary artery disease and hematological neoplasms, limits statistical power and the generalizability of the findings. Thirdly, the follow-up duration was relatively short with a wide range, which may affect the accuracy of long-term survival estimates. extended follow-up periods are warranted. Additionally, the single-group design of this study limits its capacity to assess strategy effectiveness. Different treatment strategies require comparison through rigorously designed clinical trials.

## Conclusion

This small-scale study suggests that CABG can be performed safely in most patients with stable hematological neoplasms, with no perioperative mortality. The procedure provides an opportunity for these patients to undergo further hematological treatments. The involvement of a multidisciplinary team is critical in the decision-making process. Proactive blood product transfusion and antibiotic administration are advantageous for patients in this category. However, larger cohort studies with extended follow-up are necessary to establish standardized management strategies.

## Data Availability

The raw data supporting the conclusions of this article will be made available by the authors, without undue reservation.
