# Supplementary material for: Coronary artery bypass grafting in patients with hematological neoplasms
Source: Front Cardiovasc Med. 2025 Dec 17;12:1697389. doi: 10.3389/fcvm.2025.1697389 (PMC12753907; doi:10.3389/fcvm.2025.1697389)
Supplement: Supplementary file 1 [file Supplementaryfile1.docx]

**Coronary Artery Bypass Grafting in Patients with Hematological Neoplasms**

Supplementary Table 1 distribution and status of hematological neoplasms

| Variable | Value (N=41) |
| --- | --- |
| Type |  |
| AML, n (%) | 9 (22.0) |
| ALL, n (%) | 4 (9.8) |
| CML, n (%) | 3 (7.3) |
| MDS, n (%) | 11 (26.8) |
| Lymphoma, n (%) | 5 (12.2) |
| Plasma cell neoplasms, n (%) | 2 (4.9) |
| MPN, n (%) | 7 (17.1) |
| Status |  |
| Stable, n (%) | 28 (68.3) |
| Disease-free, n (%) | 10 (24.4) |
| Progressive, n (%) | 3 (7.3) |

AML, acute myeloid leukemia; ALL, acute lymphocytic leukemia; CML, chronic myeloid leukemia; MDS, myelodysplastic syndromes; MPN, myeloproliferative neoplasms.

Supplementary Table 2 the surgical information

| Variable | Value (N=41) |
| --- | --- |
| Median sternotomy, n (%) | 15 (36.6) |
| MICS, n (%) | 26 (63.4) |
| Off-pump CABG, n (%) | 36 (87.8) |
| Vessels bypassed (number) | 2.0±1.2 |
| 1-vessel bypassed, n (%) | 20 (48.8) |
| ≥3-vessel bypassed, n (%) | 13 (31.7) |
| Use of IMA, n (%) | 39 (95.1) |
| Use of RA, n (%) | 3 (7.3) |
| Use of SVG, n (%) | 17 (41.5) |
| use of sequential grafting, n (%) | 13 (31.7) |
| use of composite conduits, n (%) | 5 (12.2) |
| Operation time (min) | 210.0 (150.0-300.0) |

Data are n (%), mean ± SD or median (IQR).

MICS, minimally invasive coronary surgery; CABG, coronary artery bypass grafting; IMA, internal mammary artery; RA, radial artery; SVG, saphenous vein graft.

Supplementary Table 3 information of laboratory test, transfusion and antibiotics

| Variable | Value (N=41) |
| --- | --- |
| Preoperative blood cell count |  |
| WBC (×10^9^/L) | 4.1 (2.6-6.6) |
| Hb (g/L) | 112.0 (82.5-132.0) |
| PLT (×10^9^/L) | 154.0 (73.0-224.5) |
| Operative Hb (g/L) | 117.0 (93.5-130.0) |
| Operative PLT (×10^9^/L) | 143.0 (65.0-224.5) |
| Blood cell count at discharge |  |
| WBC (×10^9^/L) | 5.1 (2.9-7.3) |
| Hb (g/L) | 107.0 (94.0-125.5) |
| PLT (×10^9^/L) | 170.0 (84.0-283.0) |
| Preoperative transfusion |  |
| PRBCs, n (%) | 8 (19.5) |
| platelet, n (%) | 8 (19.5) |
| FFP, n (%) | 1 (2.4) |
| Postoperative transfusion |  |
| PRBCs, n (%) | 21 (51.2) |
| platelet, n (%) | 5 (12.2) |
| FFP, n (%) | 16 (39.0) |
| Preoperative antiplatelet therapy | 30 (73.2) |
| Postoperative SAPT | 16 (39.0) |
| Postoperative DAPT | 13 (31.7) |
| No postoperative antiplatelet therapy | 12 (29.3) |
| Preoperative use of antibiotics | 8 (19.5) |

Data are n (%) or median (IQR).

WBC, white blood count; Hb, hemoglobin; PLT, platelet; PRBCs, packed red blood cells; FFP, fresh frozen plasma; SAPT, single anti-platelet therapy; DAPT, dual anti-platelet therapy.

Supplementary Table 4 information of the patients died during the follow-up

| pts | age | neoplasms | status | CABG* | postop therapy | survival (months) | cause |
| --- | --- | --- | --- | --- | --- | --- | --- |
| 1 | 63 | AML | progressive | MICS-1 | SYS | 16 | severe infection after SYS |
| 2 | 45 | AML | disease-free | MICS-1 | SYS | 12 | severe infection after SYS |
| 3 | 53 | AML | stable | MICS-1 | SYS, BMT | 11 | multiple organ failure |
| 4 | 67 | AML | progressive | MICS-3 | SYS | 40 | multiple organ failure |
| 5 | 46 | ALL | disease-free | MICS-1 | SYS | 4 | severe infection after SYS |
| 6 | 63 | MDS | stable | MICS-2 | SYS | 15 | myocardial infarction |
| 7 | 46 | MDS | stable | MICS-2 | SYS, BMT | 9 | severe infection after BMT |
| 8 | 69 | MDS | stable | Median-2 | none | 11 | cerebral hemorrhage |
| 9 | 70 | lymphoma | stable | Median-5 | SYS | 3 | multiple organ failure |

*: the number after the hypen in this column means the number of grafts.

AML, acute myeloid leukemia; ALL, acute lymphocytic leukemia; MDS, myelodysplastic syndromes; MICS, minimally invasive coronary surgery; Median, median sternotomy; SYS, systemic therapy for hematological neoplasms; BMT, bone marrow transplantation for hematological neoplasms.
